# Exosomes derived from MSC pre‐treated with oridonin alleviates myocardial IR injury by suppressing apoptosis via regulating autophagy activation

**DOI:** 10.1111/jcmm.16558

**Published:** 2021-05-06

**Authors:** Minghuan Fu, Dili Xie, Ying Sun, Yuanyuan Pan, Yunhe Zhang, Xiaohan Chen, Yong Shi, Shengnan Deng, Biao Cheng

**Affiliations:** ^1^ Department of Geriatric Cardiology Sichuan Provincial People's Hospital University of Electronic Science and Technology of China Chengdu China

**Keywords:** apoptosis, autophagy, exosomes, mesenchymal stem cells., myocardial ischaemia/reperfusion, oridonin

## Abstract

This study aimed to investigate the molecular mechanisms underlying the role of bone marrow mesenchymal stem cells (BMMSCs)‐derived exosomes in ischaemia/reperfusion (IR)‐induced damage, and the role of oridonin in the treatment of IR. Exosomes were isolated from BMMSCs. Western blot analysis was done to examine the expression of proteins including CD63, CD8, apoptotic‐linked gene product 2 interacting protein X (AliX), Beclin‐1, ATG13, B‐cell lymphoma‐2 (Bcl‐2), apoptotic peptidase activating factor 1 (Apaf1) and Bcl2‐associated X (Bax) in different treatment groups. Accordingly, the expression of CD63, CD81 and AliX was higher in BMMSCs‐EXOs and IR + BMMSCs‐EXOs + ORI groups compared with that in the BMMSCs group. And BMMSCs‐derived exosomes inhibited the progression of IR‐induced myocardial damage, while this protective effect was boosted by the pre‐treatment with oridonin. Moreover, Beclin‐1, ATG13 and Bcl‐2 were significantly down‐regulated while Apaf1 and Bax were significantly up‐regulated in IR rats. And the presence of BMMSCs‐derived exosomes partly alleviated IR‐induced dysregulation of these proteins, while the oridonin pre‐treatment boosted the effect of these BMMSCs‐derived exosomes. The inhibited proliferation and promoted apoptosis of H9c2 cells induced by hypoxia/reperfusion (HR) were mitigated by the administration of BMMSCs‐derived exosomes. Meanwhile, HR also induced down‐regulation of Beclin‐1, ATG13 and Bcl‐2 expression and up‐regulation of Apaf1 and Bax, which were mitigated by the administration of BMMSCs‐derived exosomes. And oridonin pre‐treatment boosted the effect of BMMSCs‐derived exosomes. In conclusion, our results validated that BMMSCs‐derived exosomes suppressed the IR‐induced damages by participating in the autophagy process, while the pre‐treatment with oridonin could boost the protective effect of BMMSCs‐derived exosomes.

## 1 INTRODUCTION

Myocardial ischaemia continues to be an issue of public health with high mortality and morbidity rates around the world.^1^ The major approach used to treat ischaemia is reperfusion. Scientifically, several researches have illustrated a boosted sensitivity to myocardial ischaemia/reperfusion (MIR) injuries in patients subjected to percutaneous coronary interventions or aortic bypass operations.^2^ In particular, reperfusion results in cardiovascular tissue damages, which might be much severer than ischaemia on its own and may be caused by the over‐production of reactive oxygen species, overload of intracellular Ca^2+^ as well as reperfusion arrhythmia.^1^, ^3^

Exosomes (EXOs) are one type of intracellular organelles secreted from MSCs.^4^ Recent research signifies that exosomes derived from MSCs can enhance functional recovery of spatial learning, enhance neurovascular re‐modelling such as neurogenesis as well as angiogenesis and lower the severity of neuroinflammation in post‐TBI animals.^5^, ^6^, ^7^ Since exosomes are actually believed to work as a crucial MSC mediator, the curative effects of exosomes derived from MSCs have actually been explored in detail in numerous styles of diseases, and the outcomes of these studies showed that the exosomes derived from MSCs might decrease the severity of myocardial infarctions as well as reducing IR injury‐induced hepatic damages.^8^, ^9^, ^10^ Lately, Kate et al revealed that MSCs‐Exo can induce a hepatoprotective effect.^8^ In addition, it was noted that the proliferation of hepatocytes could be promoted by MSC‐MSCs‐Exo. One other research further revealed that exosomes separated from mesenchymal stem cells promoted the proliferation of hepatocytes in liver injuries caused by carbon tetrachloride.^11^ Therefore, these results suggested that exosomes separated from MSC‐MSCs have the capacity to provide post‐injury liver protection.

Autophagy has been shown to play a crucial role in moderating IR‐induced apoptosis of cardiomyocytes.^12^ Autophagy is a process in which damaged organelles as well as proteins are degraded.^13^ Over‐activation of autophagy leads to too much self‐digestion as well as the degradation of vital cellular elements, setting off programmed cell death.^14^ In fact, it was actually proven that autophagy can be activated during the course of myocardial IR injury to result in the death of cardiomyocytes, whereas the down‐regulation of autophagy reduces the death of cardiomyocytes induced by IR injury, indicating that autophagy might be explored as a target in the therapy of myocardial IR injuries.^15^, ^16^

Oridonin is a diterpenoid separated from Rabdosia rubescens and has been reported to participate in regulation of various biological activities.^17^ Treatment with oridonin can exert various impact effects, including activating autophagic as well as regulating apoptotic signalling in a wide range of cancer cells such as colon cancer cells.^18^, ^19^, ^20^ Previous researches have also shown that numerous factors, such as ERK, Akt, FAS, NF‐κB, ROS, RTK, as well as PI3K, were associated with the anti‐tumour properties of oridonin.^20^, ^21^ It has been reported that MSCs‐derived EXOs may protect against myocardial ischaemia/reperfusion injury by activating autophagy.^22^, ^23^ Furthermore, oridonin can also enhance the activation of autophagy.^24^ In this study, we pre‐treated MSCs‐derived EXOs with oridonin to enhance the protective effect of EXOs by further activating autophagy.

## 2 MATERIALS AND METHODS

### 2.1 Animal grouping

Four groups of rat models were established in this study: (a) Sham group (N = 8; rats treated with PBS); (b) Ischaemia/reperfusion model group (termed as IR group; N = 8; rats treated to induce IR); (c) Group of IR rats treated with 10 µg/rat/d of exosomes derived from BMMSCs (termed as IR + BMMSCs‐EXOs group; N = 8); (d) Group of IR rats treated each day with 10 µg/rat/d of exosomes derived from BMMSCs that were pre‐treated with 50 µmol/L of oridonin (termed as IR +BMMSCs‐EXOs + ORI group; N = 8). The duration of treatment lasted for 7 days, and the rats were scarified to collect blood and tissue samples for subsequent analyses. Institutional ethical committee has approved the protocol of this study.

### 2.2 Isolation and culture of rat MSCs

Sprague Dawley (SD) male rats were provided by the Experimental Animal Center of our institution. The rats were 3 weeks old and weighed 80‐100 g upon arrival. Upon reception, the rats were placed in rat cages housed at 23‐24°C and were exposed to a 12 hours/12 h light‐dark cycle. All rats were given a standard chow and drinking water ad libitum. All procedures in the research were conducted according to The Guidelines for the Use of Laboratory Animals and were approved by our ethics committee. After the rats were scarified, their femora bones were collected and MSCs were separated from the bone marrow tissues collected from these rats by flushing the tissues along with a minimal essential medium (MEM, Gibco, Thermo Fisher Scientific) supplemented with 10% FBS (Gibco, Thermo Fisher Scientific) as well as suitable concentrations of streptomycin and penicillin (Sigma Aldrich). Upon centrifugation, the MSCs derived from bone marrow were pelleted and resuspended in the MEM for 7 days of culturing. The tissue culture conditions were 37°C in 5% carbon dioxide in a humidified tissue culture incubator. After the culture was ended, MSCs were identified by utilizing flow cytometry to determine the expression of MSC markers such as CD29 as well as CD44, while the expression of CD31 as well as CD34 should be absent. All antibodies required for flow cytometry, that is anti‐CD29, anti‐CD31, anti‐CD34 and anti‐CD44 primary antibodies, were purchased from Thermo Fishier Scientific and diluted in accordance with the instructions provided by the manufacturer.

### 2.3 Exosome isolation and observation with transmission electron microscopy

To investigate the potential roles of BMMSCs exosomes in IR, exosomes were extracted and observed by a transmission electron microscope. In brief, MSCs separated from 18 male rats were incubated for more than 40 hours before the exosomes in culture medium were precipitated by making use of 70 minutes of ultracentrifugation at 4°C and 100 000 *g*. Then, the purified exosomes were resuspended in saline prior to electron microscopy, during which the exosomes were fixed in 4% paraformaldehyde for 15 minutes at room temperature, pre‐coated using 0.01% Polylysine (Sigma Aldrich) and stained for 2 minutes at room using 1% phosphotungstic acid. Prior to the observation, the exosomes were also stained by using a red fluorescent dye PKH26 (Sigma Aldrich) in accordance with the instructions provided by the manufacturer. Finally, the morphological characters of separated exosomes were visualized by making use of an FEI Tecnai Sense G2 TEM (Thermo Fisher Scientific) at 50 000× magnification and 120 kV in accordance with the instructions provided by the equipment manufacturer.

### 2.4 Establishment of rat myocardial IR model and the administration of exosomes

The myocardial IR model was established using 24 male SD rats aged 7‐8 weeks, while 8 male SD rats aged 7‐8 weeks were used as the sham‐operated group. In brief, during the surgery to induce myocardial IR, the left anterior descending coronary artery in each rat was ligated. Then, after the successful establishment of the rat myocardial IR model, the exosomes were administrated into the IR rats according to a previous published method.^22^ To be specific, the rats in the IR +BMMSCs‐EXOs group and IR +BMMSCs‐EXOs +ORI group were given 10 µg/rat/d of exosomes by injection into the left ventricular wall intramyocardially. After 7 days of consecutive exosome treatment, the rats in all groups were scarified to collect blood and tissue samples for subsequent analysis.

### 2.5 Cell culture and establishment of a cellular hypoxia‐reoxygenation (H/R) model

First, to establish a H/R model in cells, rat cardiac H9c2 cells were bought from Zhong Qiao Biotech and cultured in a DMEM medium added with 10% foetal bovine serum (FBS) and 1% Penstrep (all culture media and relevant reagents were bought from Gibco, Thermo Fisher Scientific). The cell culture was done in a 5% carbon dioxide incubator at 37°C. Then, the H9c2 cells were divided into four groups that is (a) Control group (H9c2 cells treated with PBS); (b) Hypoxia/reperfusion group (H9c2 cells treated established as H/R models); (c) HR +BMMSCs‐EXOs group (H/R H9c2 cells treated with 2 µg exosomes isolated from BMMSCs); and (d) HR +BMMSCs‐EXOs +ORI group (H/R H9c2 cells treated with 2 µg exosomes isolated from BMMSCs that were pre‐treated with 50 µmol/L of ORI). The exosome concentration was chosen according to a previous publication.^22^ And the establishment of H/R models was conducted in accordance with a previous publication as well.^25^ In brief, the H9c2 cells were cultured in FBS‐deprived DMEM containing low glucose in a hypoxia condition for 4 hours and a normal oxygen condition for 24 hours. The above cell culture was done in a 5% carbon dioxide incubator. After 48 hours of treatment, the cells from all groups were harvested for subsequent analyses.

### 2.6 Study of cell proliferation using EdU *incorporation* assay

To evaluate the ability of proliferation of H9c2 cells, the cells were first cultured for 24 hours in DMEM containing 10% foetal bovine serum. Subsequently, the cells were further cultured by utilizing a serum‐free medium while EXOS derived from BMMSCs were added on top of H9c2 cells during the culture. Then, an EdU labelling reagent kit (RiboBio) was used in accordance with the instructions provided by the kit manufacturer to label the cells. After 6 hours, the cells were fixed for 30 minutes in paraformaldehyde, consequently submersed for 5 minutes in 2 mg/mL glycine and then treated with PBS containing 0.5% Triton X‐100 for twenty minutes at ambient temperature. Finally, the proliferation of H9c2 cells was assayed by using a Cell Light EdU Apollo 567 assay kit (RiboBio) in accordance with the instructions provided by the kit manufacturer and the results were evaluated by ImageJ 1.48 software (National Institutes of Health).

### 2.7 Western blot analysis

The total protein content was isolated from cell and tissue samples by using a RIPA lysis buffer (Solarbio Life Sciences) in accordance with the instructions provided by the buffer manufacturer. And the concentration of isolated protein was examined by means of a bicinchoninic acid assay kit (Yeasen Medical) in accordance with the instructions provided by the kit manufacturer. Then, 15 µg of total protein isolated from each sample was resolved by using 10% polyacrylamide gel electrophoresis, and the resolved protein was blotted to polyvinylidene fluoride membranes (Merck Millipore), which were then blocked for one hour in 5% bovine serum albumin at ambient temperature before being incubated using primary anti‐CD63, anti‐CD8, anti‐AliX, anti‐Beclin‐1, anti‐ATG13, anti‐Bax, anti‐Bcl‐2 and anti‐Apaf1 antibodies as well as suitable horseradish peroxidase‐tagged secondary antibodies (Abcam) in accordance with the instructions provided by the antibody manufacturer. Finally, after colour development using an ECL reagent (Merck Millipore) in accordance with the instructions provided by the reagent manufacturer, the relative protein expression of CD63, CD8, AliX, Beclin‐1, ATG13, Bcl‐2, Apaf1 and Bax in each sample was quantified by ImageJ 1.48 software (National Institutes of Health).

### 2.8 Study of apoptosis of myocardial tissues using TUNEL assay

The apoptosis of cells collected from myocardial tissues was examined by using a terminal deoxynucleotidyl transferase dUTP nick end labelling (TUNEL) staining kit (Roche)in accordance with the instructions provided by the kit manufacturer. The presence of apoptotic cells was observed and captured on film by using an IX53 optical microscope (Olympus).

### 2.9 Study of myocardial injury

To study the status of myocardial injury in each group of rats, collected myocardial tissues were embedded in paraffin, sliced into 4 µm thick sections (including cross section and longitudinal section) and then stained by using an haematoxylin and eosin (H&E) staining kit (RiboBio) and Masson's trichrome staining in accordance with the instructions provided by the kit manufacturer.

### 2.10 Statistical analysis

All results were represented by mean  ± standard deviations. SPSS software version 22.0 (SPSS, IBM, Chicago, IL) was used to carry out all statistical analysis. All experimental procedures were repeated for a minimum of 3 times. Student's *t* tests and one‐way ANOVA were utilized for statistical comparisons when appropriate. A *P* value of < .05 was deemed statistically significant.

## 3 RESULTS

### 3.1 Isolation of exosomes from BMMSCs

**FIGURE 1:**
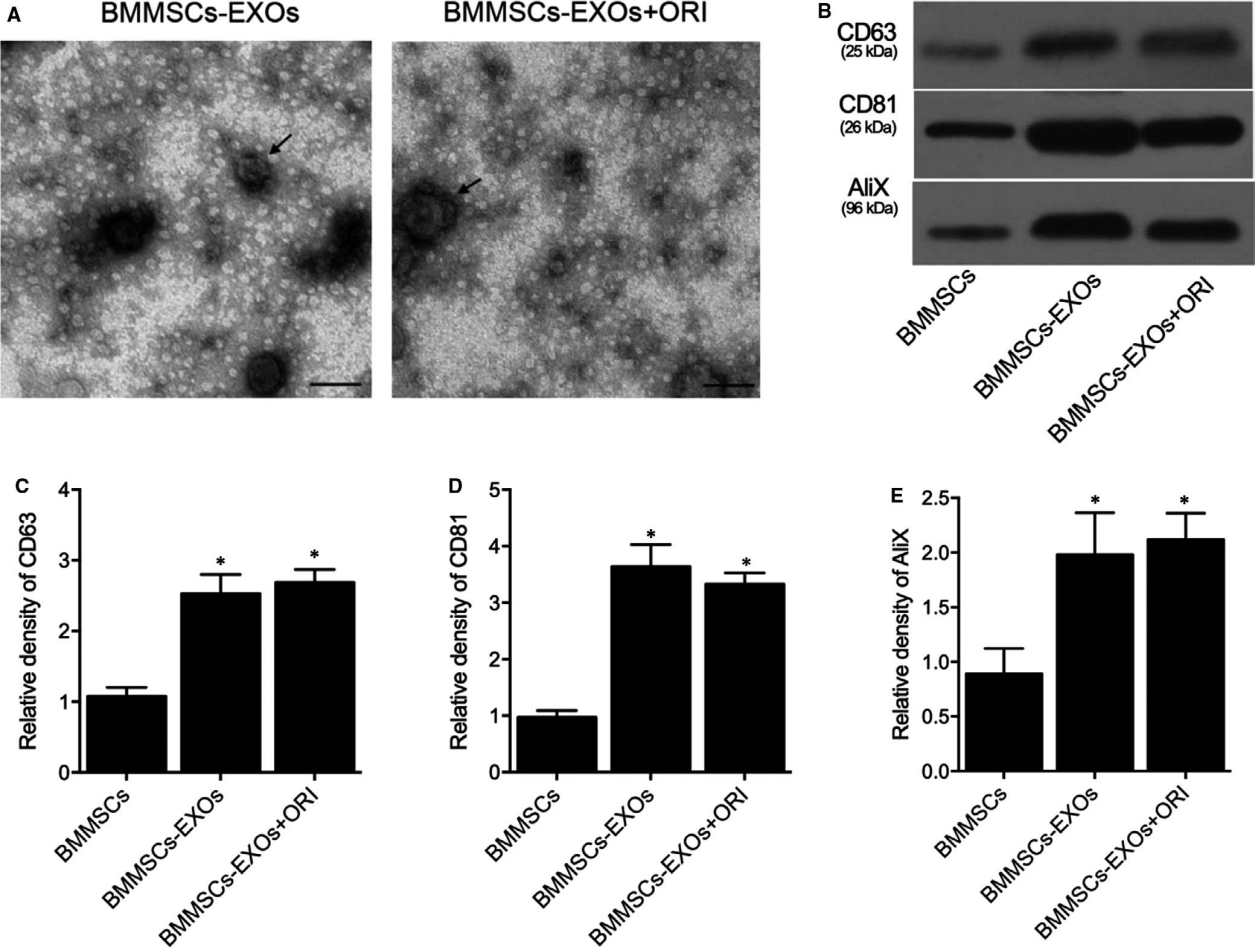
Exosomes isolated from BMMSCs were visualized by a transmission electron microscope, and the expression of CD63, CD81 and AliX was compared among the BMMSCs, BMMSCs‐EXOs and BMMSCs‐EXOs + ORI groups (n = 3; *P value < .05 vs BMMSCs group). A, The general shape of exosomes isolated from BMMSCs‐EXOs group and BMMSCs‐EXOs + ORI group; B, Western blot of CD63, CD81 and AliX expression in BMMSCs, BMMSCs‐EXOs and BMMSCs‐EXOs + ORI groups; C, Relative density of CD63 in BMMSCs, BMMSCs‐EXOs and BMMSCs‐EXOs + ORI groups; D, Relative density of CD81 in BMMSCs, BMMSCs‐EXOs and BMMSCs‐EXOs + ORI groups; E, Relative density of AliX in BMMSCs, BMMSCs‐EXOs and BMMSCs‐EXOs + ORI groups

Exosomes were extracted from BMMSCs and observed by a transmission electron microscope, and the typical shape of isolated exosomes was shown in Figure 1A. We also compared the expression of cell surface proteins, including CD63, CD81 and AliX, in the BMMSCs group, BMMSCs‐EXOs group and BMMSCs‐EXOs + ORI group. As shown by the protein bands obtained by Western blot analysis, the expression of CD63 (Figure 1B,C), CD81 (Figure 1B,D) and AliX (Figure 1B,E) in BMMSCs‐EXOs and BMMSCs‐EXOs + ORI groups was higher than in the BMMSCs group.

### 3.2 IR‐induced myocardial damage was suppressed by BMMSCs‑derived exosomes

**FIGURE 2:**
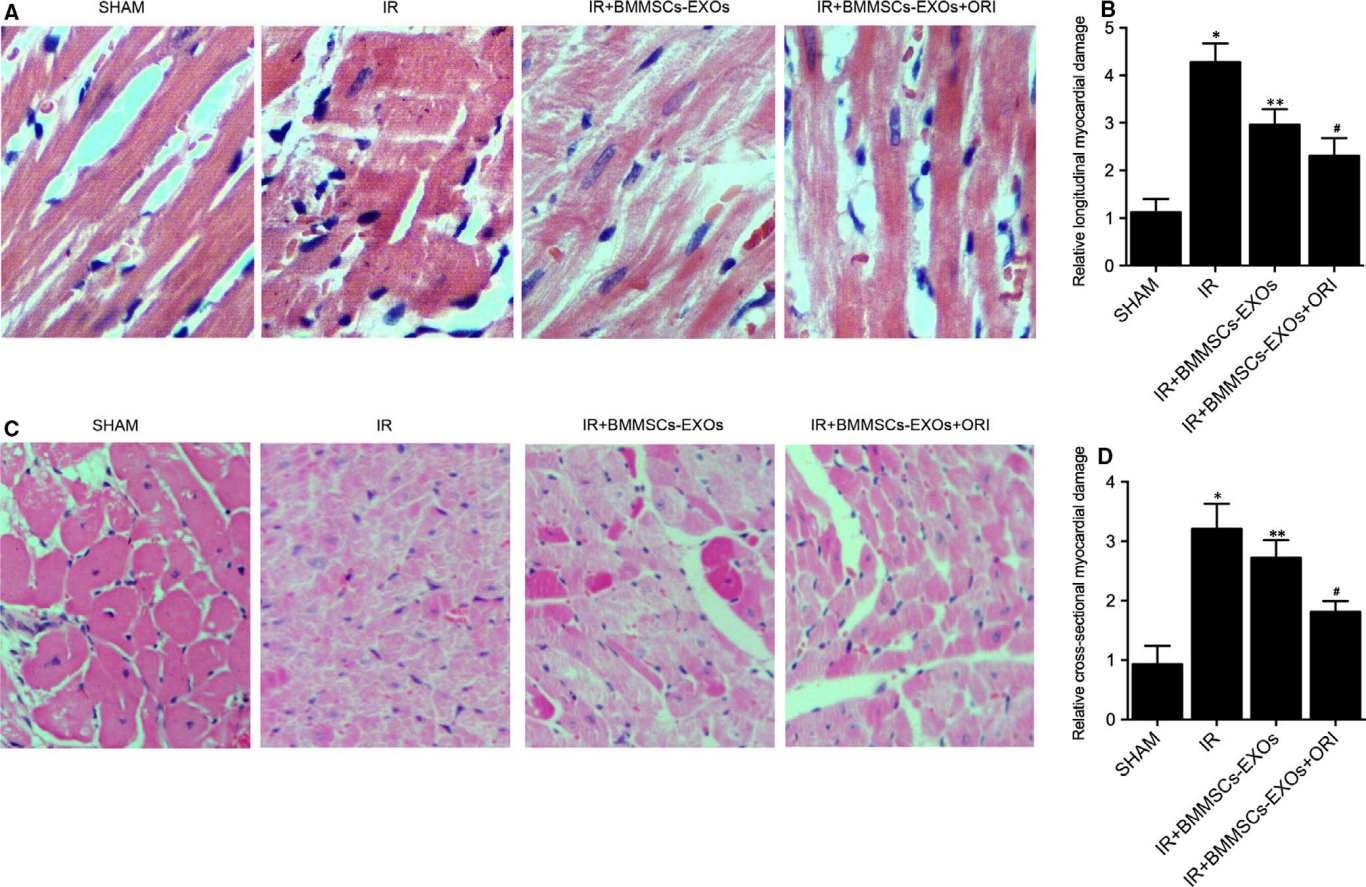
H&E staining indicated that BMMSCs‐derived exosomes inhibited the progression of IR‐induced myocardial damage in IR + BMMSCs‐EXOs and IR + BMMSCs‐EXOs + ORI groups, and the pre‐treatment with oridonin enhanced the effect of BMMSCs‐derived exosomes. A, H&E staining results of longitudinal section of myocardial tissues collected from the sham group, IR group, IR + BMMSCs‐EXOs group and IR + BMMSCs‐EXOs + ORI group; B, Quantitative H&E staining results of longitudinal myocardial tissues in different rat groups; C, H&E staining results of cross section of myocardial tissues collected from the sham group, IR group, IR + BMMSCs‐EXOs group and IR + BMMSCs‐EXOs + ORI group; D, Quantitative H&E staining results of cross‐sectional myocardial tissues in different rat groups

**FIGURE 3:**
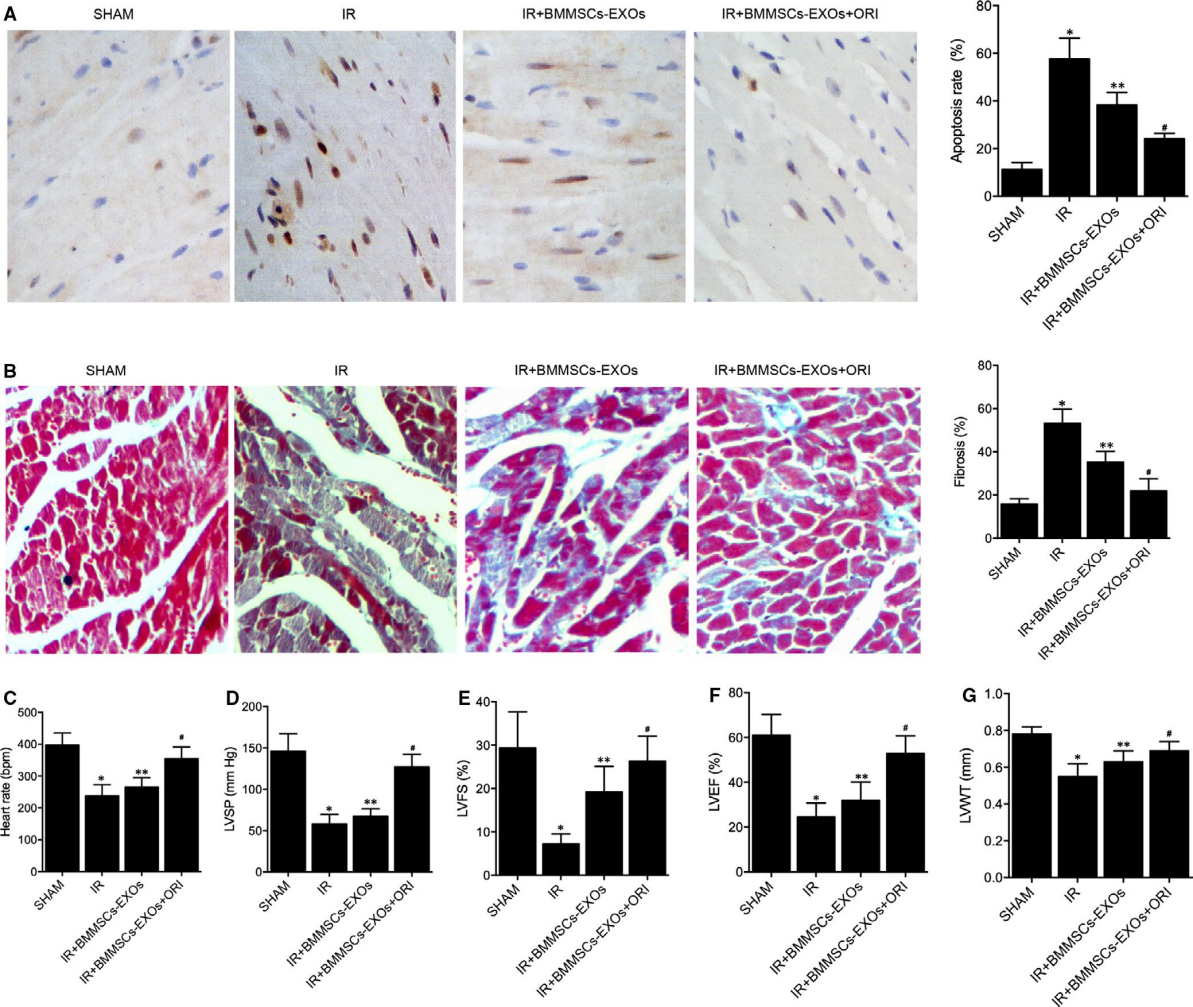
TUNEL staining and Masson's trichrome staining indicated that IR‐induced apoptosis and fibrosis, as well as damaged cardiac functions, were restored by BMMSCs‐derived exosomes in IR + BMMSCs‐EXOs and IR + BMMSCs‐EXOs + ORI groups, and the pre‐treatment with oridonin enhanced the effect of BMMSCs‐derived exosomes (n = 3; *P value < .05 vs SHAM group; **P value .05 vs IR group; ^#^ *P* value < .05 vs IR + BMMSCs‐EXOs group). A, TUNEL staining of IR‐induced apoptosis of myocardial tissues collected from the sham group, IR group, IR + BMMSCs‐EXOs group and IR + BMMSCs‐EXOs + ORI group (Brown indicates TUNEL positive cells, and blue indicates TUNEL negative cells); B, Masson's trichrome staining of IR‐induced fibrosis of myocardial tissues collected from the sham group, IR group, IR + BMMSCs‐EXOs group and IR + BMMSCs‐EXOs + ORI group (Blue indicate fibrosis); C, Heart rate of the sham group, IR group, IR + BMMSCs‐EXOs group and IR + BMMSCs‐EXOs + ORI group; D, LVSP of the sham group, IR group, IR + BMMSCs‐EXOs group and IR + BMMSCs‐EXOs + ORI group; E, LVFS of the sham group, IR group, IR + BMMSCs‐EXOs group and IR + BMMSCs‐EXOs + ORI group; F, LVEF of the sham group, IR group, IR + BMMSCs‐EXOs group and IR + BMMSCs‐EXOs + ORI group; G, LVWT of the sham group, IR group, IR + BMMSCs‐EXOs group and IR + BMMSCs‐EXOs + ORI group

Four groups of rats were established: (a) Sham group; (b) IR group; (c) IR + BMMSCs‐EXOs group; (d) IR + BMMSCs‐EXOs + ORI group. H&E and TUNEL assays were conducted to observe the effect of BMMSCs‐derived exosomes on IR‐induced myocardial damage. As shown in Figure 2, the H&E staining of longitudinal section (Figure 2A,B) or cross section (Figure 2C,D) of myocardial tissues indicated that the application of BMMSCs‐derived exosomes inhibited the progression of IR‐induced myocardial damage in IR + BMMSCs‐EXOs and IR + BMMSCs‐EXOs + ORI groups, and the protective effect of BMMSCs‐derived exosomes was boosted by pre‐treatment with oridonin. However, although the abnormal cellular appearance was mitigated by the administration of BMMSCs‐derived exosomes, the irregular cell shape and lining in the myocardium of rats in IR groups were still more visible compared with that in the sham group. Subsequently, as shown in Figure 3A, the TUNEL staining assay resulted in a significant increase in the number of apoptotic cells was presented in the IR group, and the number of apoptotic cells was reduced by BMMSCs‐derived exosomes, with ORI treatment inducing a more impressive effect. Similarly, the changes of fibrosis detected by Masson's trichrome staining (Figure 3B) also showed the same tendency as the apoptosis rate. Furthermore, when observing the effect of BMMSCs‐derived exosomes upon cardiac functions, parameters including heart rate (Figure 3C), LVSP (Figure 3D), LVFS (Figure 3E), LVEF (Figure 3F) and LVWT (Figure 3G) were all elevated in IR rats, while the administration of BMMSCs‐derived exosomes restored the down‐regulation of these cardiac parameters. And it is noteworthy that the pre‐treatment of oridonin also boosted the effect of BMMSCs‐derived exosomes upon cardiac functions.

### 3.3 The dysregulation of Beclin‐1, ATG13, Apaf1, Bcl‐2 and Bax expression was reversed by BMMSCs‑derived exosomes in vivo

**FIGURE 4:**
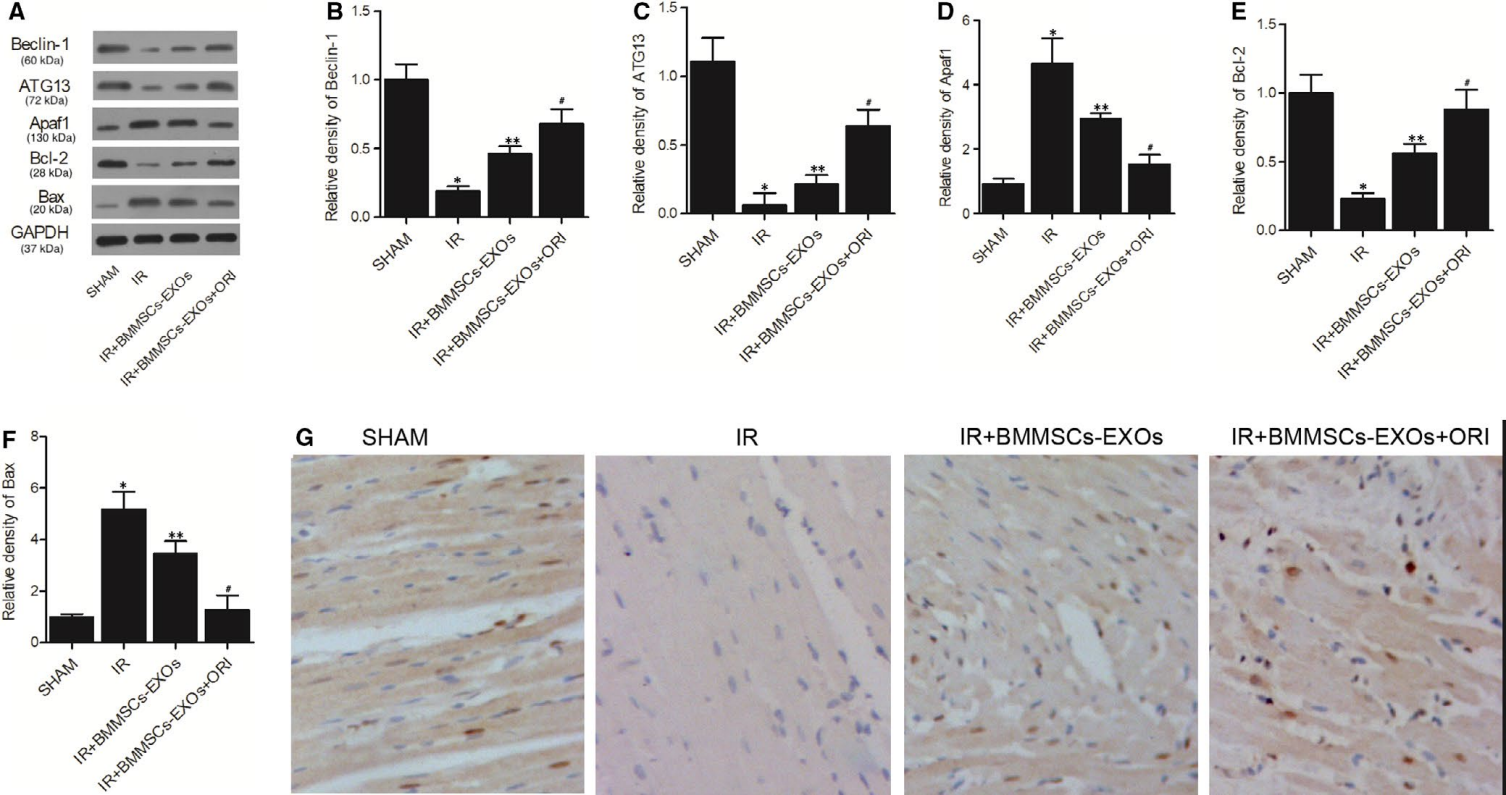
The dysregulation of Beclin‐1, ATG13, Apaf1, Bcl‐2, Bax and Ki67 expression was reversed by BMMSCs‑derived exosomes in vivo (n = 3; *P value < .05 vs SHAM group; **P value .05 vs IR group; ^#^ *P* value < .05 vs IR + BMMSCs‐EXOs group). A, Western blot of Beclin‐1, ATG13 and Apaf1 expression in the sham group, IR group, IR + BMMSCs‐EXOs group and IR + BMMSCs‐EXOs + ORI group; B, Protein expression of Beclin‐1 in the sham group, IR group, IR + BMMSCs‐EXOs group and IR + BMMSCs‐EXOs + ORI group; C, Protein expression of ATG13 in the sham group, IR group, IR + BMMSCs‐EXOs group and IR + BMMSCs‐EXOs + ORI group; D, Protein expression of Apaf1 in the sham group, IR group, IR + BMMSCs‐EXOs group and IR + BMMSCs‐EXOs + ORI group; E, Protein expression of Bcl‐2 in the sham group, IR group, IR + BMMSCs‐EXOs group and IR + BMMSCs‐EXOs + ORI group; F, Protein expression of Bax in the sham group, IR group, IR + BMMSCs‐EXOs group and IR + BMMSCs‐EXOs + ORI group; G, IHC assay of Ki67 in the sham group, IR group, IR + BMMSCs‐EXOs group and IR + BMMSCs‐EXOs + ORI group

Western blot was performed to observe the effect of BMMSCs‐derived exosomes on the expression of Beclin‐1, ATG13 and Apaf1 in vivo. As indicated by Western blot results, the expression of Beclin‐1 (Figure 4A,B), ATG13 (Figure 4A,C) and Bcl‐2 (Figure 4A,4E) was significantly down‐regulated in IR rats. However, IR‐induced down‐regulation of Beclin‐1, ATG13 and Bcl‐2 expression was mitigated by the administration of BMMSCs‐derived exosomes, and the pre‐treatment with oridonin increased the effect of BMMSCs‐derived exosomes. Meanwhile, the levels of Apaf1 (Figure 4A,D) and Bax (Figure 4A,F) were the highest in the IR group, while the presence of BMMSCs‐derived exosomes partly reduced IR‐induced up‐regulation in Apaf1 and Bax expression. Moreover, as shown in Figure 4G, the IHC assay of Ki67 also indicated that the significant down‐regulation of Ki67 in IR rats was obstructed by the administration of BMMSCs‐derived exosomes while the pre‐treatment of oridonin boosted the effect of these exosomes.

### 3.4 Administration of BMMSCs‐derived exosomes affected the proliferation and apoptosis of H9c2 cells

**FIGURE 5:**
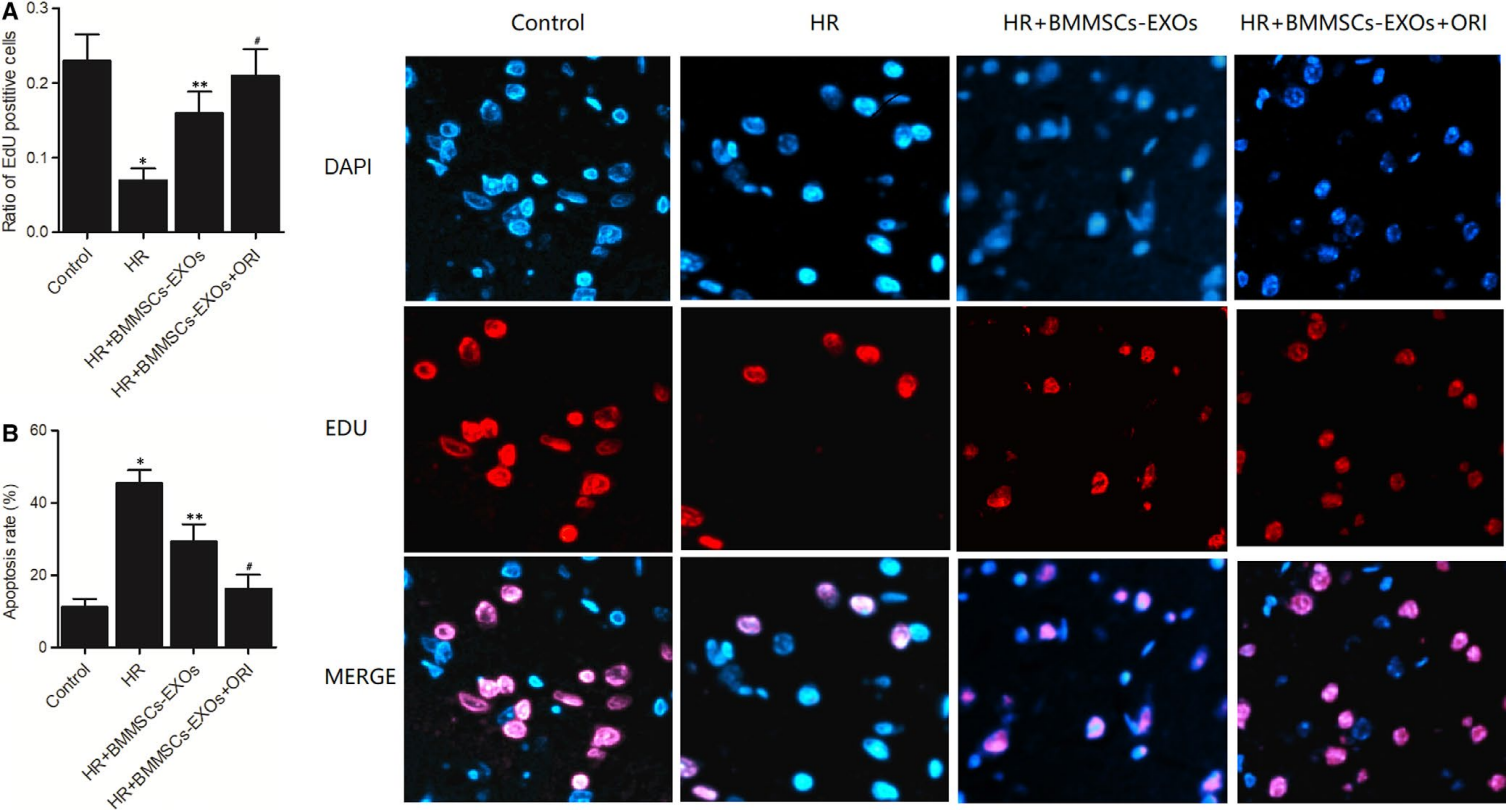
Administration of BMMSCs‐derived exosomes affected the proliferation and apoptosis of H9c2 cells (*P value < .05 vs control group; **P value .05 vs HR group; ^#^ *P* value < .05 vs HR + BMMSCs‐EXOs group). A, Ratio of EdU positive cells in the control group, HR group, HR + BMMSCs‐EXOs group and HR + BMMSCs‐EXOs + ORI group; B, Apoptosis rate of H9c2 cells in the control group, HR group, HR + BMMSCs‐EXOs group and HR + BMMSCs‐EXOs + ORI group

Four groups of H9c2 cells were set up that is (a) Control group; (b) HR group; (c) HR + BMMSCs‐EXOs group; 4. HR + BMMSCs‐EXOs + ORI group. We observed the proliferation and apoptosis of H9c2 cells. As shown in Figure 5A, the ratio of EdU positive cells was decreased in the HR group and the treatment with BMMSCs‐derived exosomes increased the ratio of EdU positive cells, and oridonin pre‐treatment increased the effect of BMMSCs‐derived exosomes. Also, as shown in Figure 5B, the apoptosis rate of H9c2 cells was the highest in the HR group and the lowest in the control group.

### 3.5 The dysregulation of Beclin‐1, ATG13, Apaf1, Bcl‐2 and Bax expression was reversed by BMMSCs‑derived exosomes in vitro

**FIGURE 6:**
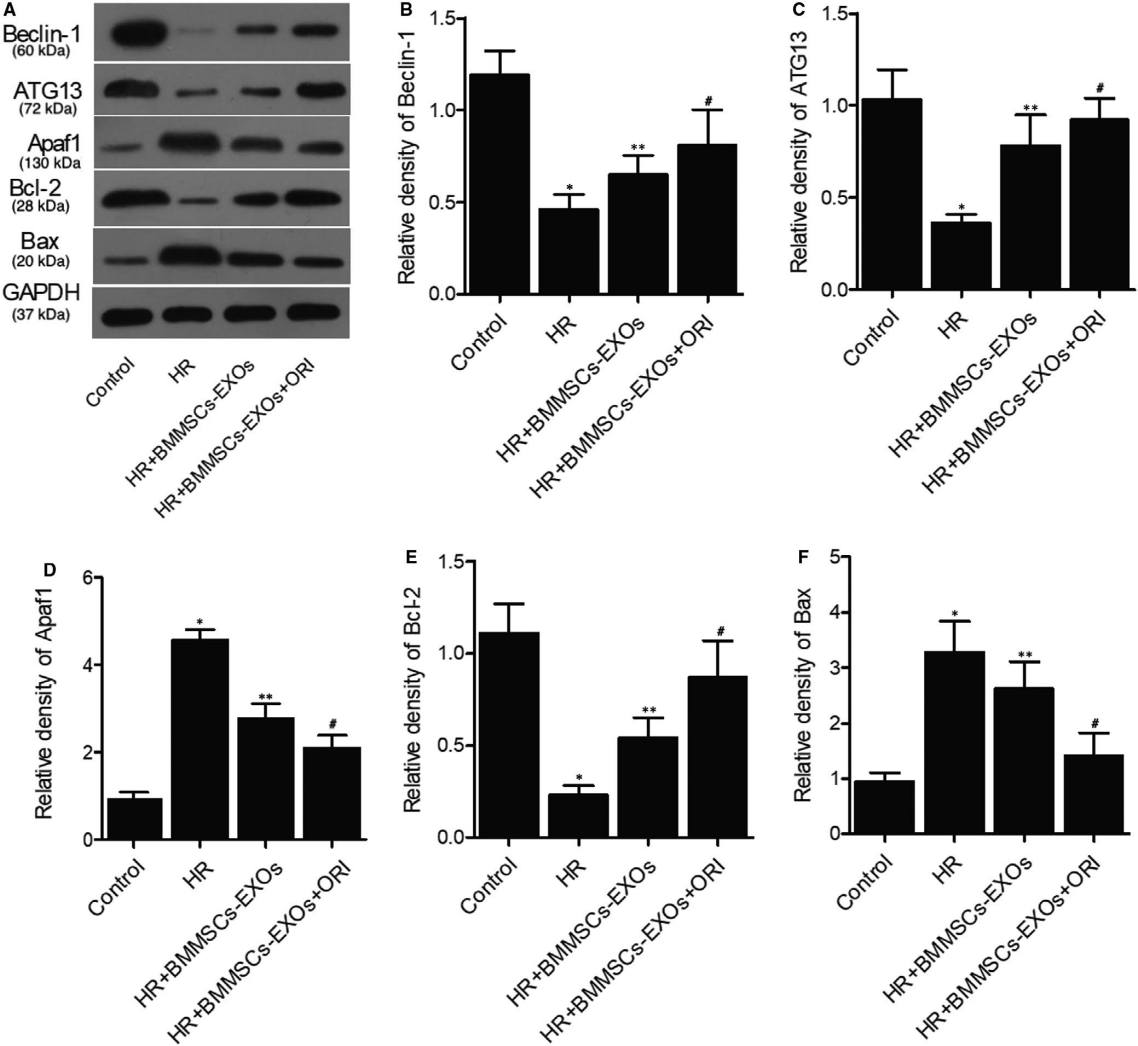
The dysregulation of Beclin‐1, ATG13, Apaf1, Bcl‐2 and Bax expression was reversed by BMMSCs‑derived exosomes in vitro (*P value < .05 vs control group; **P value .05 vs HR group; ^#^ *P* value < .05 vs HR + BMMSCs‐EXOs group). A, Western blot of Beclin‐1, ATG13 and Apaf1 expression in the control group, HR group, HR + BMMSCs‐EXOs group and HR + BMMSCs‐EXOs + ORI group; B, Protein expression of Beclin‐1 in the control group, HR group, HR + BMMSCs‐EXOs group and HR + BMMSCs‐EXOs + ORI group; C, Protein expression of ATG13 in the control group, HR group, HR + BMMSCs‐EXOs group and HR + BMMSCs‐EXOs + ORI group; D, Protein expression of Apaf1 in the control group, HR group, HR + BMMSCs‐EXOs group and HR + BMMSCs‐EXOs + ORI group; E, Protein expression of Bcl‐2 in the control group, HR group, HR + BMMSCs‐EXOs group and HR + BMMSCs‐EXOs + ORI group; F, Protein expression of Bax in the control group, HR group, HR + BMMSCs‐EXOs group and HR + BMMSCs‐EXOs + ORI group

Western blot was performed to observe the effect of BMMSCs‐derived exosomes on the expression of Beclin‐1, ATG13, Apaf1, Bcl‐2 and Bax in vitro. Accordingly, HR down‐regulated the expression of Beclin‐1 (Figure 6A,B), ATG13 (Figure 6A,C) and Bcl‐2 (Figure 6A,E) in H9c2 cells, and the effect of HR was mitigated by the administration of BMMSCs‐derived exosomes. Moreover, pre‐treatment of BMMSCs‐derived exosomes with oridonin promoted the effect of BMMSCs‐derived exosomes on the expression of above proteins. Meanwhile, the presence of BMMSCs‐derived exosomes in HR + BMMSCs‐EXOs and HR + BMMSCs‐EXOs + ORI groups partly reduced HR‐induced up‐regulation of Apaf1 (Figure 6A,D) and Bax (Figure 6A,F) expression in H9c2 cells.

## 4 DISCUSSION

Autophagy was shown to be associated with myocardial IR injuries. In addition, berbamine can protect heart tissues against IR injuries by inhibiting IR‐induced impairment in the processing of autophagosomes in cardiomyocytes, thus increasing the levels of LC3‐II as well as Beclin1.^26^ In addition, it was suggested that autophagy was associated with a protective effect against myocardial IR injuries.^27^ Furthermore, IR can cause inflammatory reactions, and autophagy acts as a survival mechanism in IR cells by enhancing the synthesis of IL‐6.^28^ Therefore, the level of autophagy is linked to the likelihood of cell survival. On the other hand, if we can inhibit the process of autophagy in cancer cells, we may render these cells more sensitive to medications like chloroquine.^29^ The application of BMMSCs‐derived exosomes inhibited the progression of IR‐induced myocardial damage. Moreover, apart from exosomes, some other cell‐based therapeutic approaches have been investigated. For example, it has been reported that the pre‐vascularized cardiac stromal cell patch generated from therapeutic cardiosphere‐derived stromal cells integrated with engineered micro‐vessels evidently improved the process of recovery from myocardial infarction in animal models.^30^, ^31^ Moreover, other methods including bispecific antibody inhalation therapy, platelet‐inspired nanocell injection and nanogel‐encapsulated human cardiac stem cell injection were also reported.^32^, ^33^, ^34^, ^35^

Exosomes derived from MSC were first studied in mice with myocardial IR injuries.^36^ MSCs were actually shown to generate a much higher level of exosomes than other types of cells including myoblasts as well as embryonic kidney cells.^37^ There is actually no difference between exosomes isolated from MSCs and other types of cells regarding their morphology as well as storage conditions. Regarding the identification of exosomes, exosomes derived from MSCs not only express usual exosome markers including CD9 as well as CD81, but also express additional adhesion molecules such as CD29, CD73 as well as CD44 on the cell membrane.^38^ Numerous studies have actually illustrated that a number of different types of exosomes derived from stem cells have the capacity to strengthen overall heart functions while attenuating ventricular re‐modelling by means of suppressing stress‐induced apoptosis as well as by boosting angiogenesis in myocardial IR injuries.^39^, ^40^ Nonetheless, the exact impact of MSC‐Exo on inflammation of myocardial IR injuries remains unknown, although some research showed that MSC‐Exo strongly changed the state of polarization of macrophages in mice with myocardial IR injuries.^41^ In this study, we found that the expression of Beclin‐1, ATG13 and Bcl‐2 was significantly down‐regulated while the expression of Apaf1 and Bax was significantly up‐regulated in IR rats. Accordingly, the presence of BMMSCs‐derived exosomes partly alleviated IR‐induced dysregulation of these proteins, with the pre‐treatment by oridonin boosting the effect of BMMSCs‐derived exosomes. It was actually shown that an improved level of autophagy might be used as a unique approach to strengthen the viability of hepatocytes in IR‐induced injuries, while the impact of autophagy might be linked to its anti‐inflammatory as well as anti‐apoptotic features.^42^

Oridonin is a bioactive diterpenoid present in Rabdosia rubescens and has actually been commonly utilized in traditional Chinese medicines.^43^ Oridonin has demonstrated great anti‐cancer features including the arrest of cell cycles, the induction of apoptosis, as well as the suppression of angiogenesis.^44^ Furthermore, Rabdosia rubescens as well as Oridonin demonstrated anti‐inflammatory features and have been used as natural medication for the therapy of inflammatory conditions by suppressing MAPK or NF‐κB activation to subdue the secretion of pro‐inflammatory cytokines, including TNF‐α as well as IL‐6.^45^, ^46^, ^47^ In this study, we found that the ratio of EdU positive H9c2 cells was decreased in the HR group while their apoptosis rate was increased. The treatment by BMMSCs‐derived exosomes mitigated the above situation, and the pre‐treatment with oridonin promoted the effect of BMMSCs‐derived exosomes.

ECG results indicated successful establishment of an MIR injury model, and Oridonin could mitigate MIR injury‐induced elevation in the ST segment.^48^ Previous researches suggested that oridonin may exert its effect via moderating the activation of autophagy, thus supplying ATP while preventing the production of reactive oxygen species.^21^, ^49^, ^50^ On the other hand, medicinal treatments targeting the pathway of autophagosome‐lysosome relieved the severity of cardiac remodelling.^24^, ^51^ In this study, we found that HR down‐regulated the expression of Beclin‐1, ATG13 and Bcl‐2, and up‐regulated the expression of Apaf1 and Bax. The administration of BMMSCs‐derived exosomes partly recovered the normal expression of above proteins, and the pre‐treatment with oridonin boosted the effect of BMMSCs‐derived exosomes.

## 5 CONCLUSION

The findings of this study demonstrated that the administration of BMMSCs‐derived exosomes protected myocardiocytes against ischaemia reperfusion injury by suppressing apoptosis and promoting autophagy, and the pre‐treatment with oridonin enhanced the protective effect of BMMSCs‐derived exosomes by further promoting autophagy activation.

## CONFLICT OF INTEREST

None.

## AUTHOR CONTRIBUTIONS

**Minghuan Fu:** Conceptualization (equal); Investigation (equal); Methodology (equal); Project administration (equal); Software (equal); Supervision (equal). **Dili Xie:** Conceptualization (equal); Funding acquisition (equal); Investigation (equal); Methodology (equal); Supervision (equal); Validation (equal). **Ying Sun:** Investigation (equal); Methodology (equal); Resources (equal). **Yuanyuan Pan:** Formal analysis (equal); Investigation (equal); Resources (equal); Validation (equal). **Yunhe Zhang:** Investigation (equal); Software (equal). **Xiaohan Chen:** Investigation (equal); Validation (equal); Writing‐review & editing (equal). **Yong Shi:** Investigation (equal); Visualization (equal). **Shengnan Deng:** Validation (equal); Visualization (equal); Writing‐original draft (equal). **Biao Cheng:** Conceptualization (equal); Data curation (equal); Formal analysis (equal); Resources (equal); Software (equal); Supervision (equal); Validation (equal); Writing‐original draft (equal).

## ETHICAL APPROVAL

Not applicable.

## CONSENT FOR PUBLICATION

Not applicable.

## Data Availability

The data that support the findings of this study are available from the corresponding author upon reasonable request.
